# Editorial: Metacognitive Therapy: Science and Practice of a Paradigm

**DOI:** 10.3389/fpsyg.2020.576210

**Published:** 2020-09-18

**Authors:** Adrian Wells, Lora Capobianco, Gerald Matthews, Hans M. Nordahl

**Affiliations:** ^1^Faculty of Biology Medicine and Health, School of Psychological Sciences, University of Manchester, Manchester, United Kingdom; ^2^Research and Innovation, Greater Manchester Mental Health National Health Service Foundation Trust, Manchester, United Kingdom; ^3^Institute for Simulation and Training, University of Central Florida, Orlando, FL, United States; ^4^Department of Mental Health, Norwegian University of Science and Technology, Trondheim, Norway

**Keywords:** metacognitive therapy, S-REF model, metacognition, psychological disorders, causal mechanisms

One of the greatest challenges facing mental health research is the development and testing of bone-fide causal theories of psychopathology that inform the development of more effective treatments. Unfortunately, apart from the major progress offered by cognitive-behavior therapy over 40 years ago, there have been few advances in models and treatments that have improved outcomes. Developments are hindered by the prevailing clinical research strategy that has attempted to innovate by combining therapeutic techniques taken from a wide range of existing sources, but in the absence of an understanding of causal mechanisms. This raises crucial questions: how can the researcher or practitioner know which of the plethora of techniques to choose, should they be combined or used in the absence of a theoretical rationale and are they compatible?

It is evident that progress could be made by developing a more rigorous, scientifically grounded theory of causal mechanisms, and devising treatment techniques ground-up from this theoretical platform. This approach was used by Wells and Matthews (1994), (see also Wells and Matthews, 2015) in the development of their S-REF model, which offered the early foundations of metacognitive therapy (MCT) (Wells, 2009, 2019) based on the cognitive science of emotion. The present Research Topic aims to capture the breadth of current ideas and studies in MCT and bring together active researchers at the forefront of the field. The objectives are to demonstrate the universal influence of MCT, present data probing theoretical mechanisms and to offer a grounding from which ideas can spring that will support future investigations.

There are 30 articles in this issue covering advanced theory, evaluation of mechanisms, clinical evaluations of treatment efficacy, feasibility of novel applications of treatment and studies of assessment tools. The articles consist of clinical, non-clinical, cognitive and neuroscience studies, research in adults and children, and studies of personality, stress, psychosis, alcohol abuse, anxiety, trauma, obsessions and depression. The articles are grouped into two clusters. First, work on theory and mechanisms is presented and this is followed by studies of the clinical effects of MCT.

MCT is based on some basic principles central to the S-REF model: (1) most disorders are caused by a common or transdiagnostic set of processes made up of difficult to control extended negative thinking, (2) psychological distress is prone to self-correct but is thwarted in doing so by maladaptive self-regulatory strategies, (3) metacognitions are key to adaptive and maladaptive self-regulatory processes.

Wells elaborates on the original S-REF model and makes important and more detailed distinctions between cognitive and metacognitive structures and processes, drawing out the necessary components and hypothesized circuits in formulating adaptive and maladaptive self-regulation. The paper describes a metacognitive control system involved in psychological disorders and elucidates different types of metacognitive information that influence the way cognition is experienced. Repetitive and extended negative processing that maintains psychological distress is a process normally prone to decay but this is thwarted by maladaption in the metacognitive system leading to persistence of negative processing. The model leads to predictions of the existence of several important mechanisms and types of metacognitive information including “cybernetic code” generated by the metacognitive system that impact on neural networks and contribute to emotional recovery or sustained processing and psychological disorder. The model is broadened to consider how metacognitive information and its flow between systems helps to create embodiment, self-awareness and meta-representational states that provide resources for self-regulation. The paper concludes by exploring how the model has shaped the development and focus of MCT and explores the implications for future treatment development and advances in theory and research.

Concepts of neuroticism and trait-anxiety are widely used to measure psychological vulnerability. Nevertheless, they can be limiting because they do not identify the underlying mechanisms of disorder; instead, they focus on the likelihood of experiencing symptoms. Nordahl et al. show that negative and positive beliefs about worry are both cross sectional and prospective predictors of trait-anxiety, suggesting that dysfunction in metacognition might be the underlying mechanism that is captured by emotion-vulnerability measures. The direction of causality in metacognition-emotion relationships is addressed by Capobianco et al. using cross-lagged structural equation modeling. They found that metacognitive beliefs predicted subsequent anxiety and anxiety predicted subsequent metacognition over different time-courses suggesting mutual causal links that might (if measured over a longer time-frame) constitute a dysfunctional metacognition-emotion cycle. One important way to examine emotional vulnerability is to assess multiple traits that contribute not only to dysfunction but also those that may confer the opposite; psychological resilience. Matthews et al. examined the effects of meta-worry, worry and resilience traits on the performance of a complex task under two types of stressor, differing in self-reference. Meta-worry was associated with subjective stress and EEG responses to the more self-referent stressor (negative feedback). Moderator effects on associations between state worry, performance and EEG measures suggested that high trait meta-worry blocks adaptation to stress through compensatory effort.

The Metacognitions Questionnaire (MCQ) is the most commonly used measure of adult metacognitive beliefs linked to disorder in the metacognitive theory. The MCQ has also been adjusted for use in children and adolescents as reviewed in this special issue by Myers et al. These authors examined the psychometric properties of variants of the MCQ, demonstrating a similar latent structure, reliability, and validity estimates in child versions to those obtained in the original scale. Furthermore, theoretically expected relationships between metacognitions and emotion disorder symptoms are evident, resembling those found in adults.

Utilizing the child version of the MCQ, Reinholdt-Dunne et al. demonstrated elevated dysfunctional metacognitions and lower self-report attentional control in a clinical compared with a community sample of 7–14 year-olds. In the community but not the clinical sample, MCQ-total interacted with attentional control in explaining symptoms of anxiety. The result is consistent with the idea that detrimental effects of metacognitive beliefs might be remediated by high-levels of perceived attention control.

Fergus et al. specifically examined the effect of metacognitive beliefs on mental contamination (feelings of internal dirtiness) in women who had experienced sexual trauma. Following exposure to an evoking stimulus, metacognitions concerning uncontrollability and danger, low cognitive confidence, and need to control thoughts positively correlated with the severity of mental contamination. The strength of relationship between specific metacognitive beliefs and symptoms of psychological disorder is likely to be subject to a range of other metacognitive influences as specified in the MCT model. Bardeen and Fergus examined this issue in the context of PTSD symptoms. They found that amongst adults exposed to trauma, deficits in executive control strengthened the positive association between positive metacognitive beliefs (e.g., “worrying will keep me safe”) and PTSD symptom scores. The positive relationship between negative metacognitive beliefs and symptoms was not moderated by executive control.

Two articles in this special issue specifically examine the Cognitive Attentional Syndrome (CAS) defined as a combination of repetitive negative thinking, unhelpful coping strategies and underlying dysfunctional metacognitions. In one of these studies, Faija et al. report on the Cognitive Attentional Syndrome Scale-1 (CAS-1) adapted for research in cardiac rehabilitation patients reporting anxiety and depression. A three-factor solution was supported by confirmatory factor analysis composed of coping strategies, negative, and positive metacognitive beliefs. Each subscale independently contributed to anxiety while coping strategies independently contributed to depression symptoms.

One way to objectively validate the effects of the CAS as proposed in the S-REF model is by testing for specific neural correlates of this syndrome, a task undertaken by Kowalski et al. Their study explored the neural correlates of the CAS using fMRI during induced negative thinking. Low- and high-CAS groups differed in functional connectivity during induced negative and abstract thinking and also in resting state fMRI. The results suggest disrupted self-referential processing in individuals who score high on self-report CAS dimensions.

Three articles report on laboratory-based effects of an individual MCT treatment technique; the Attention Training (ATT). ATT was developed to attenuate the CAS, by reducing self-focused processing and strengthening knowledge concerning flexible control of thinking. Knowles and Wells demonstrated that a single session of the ATT increased resting alpha and beta oscillations in front-parietal brain regions when compared with a control condition. The signature and location of effects is consistent with the ATT affecting executive control processes for which it was designed. Continuing with the evaluation of objective ATT effects. Barth et al. tested effects on attentional performance across attention bias, inhibition, working memory and disengagement tasks. The results showed specific effects on attention bias suggesting that ATT might promote greater attention flexibility in healthy subjects. In a related paper, Heitland et al. tested whether pre-treatment attentional control was related to these effects of ATT. Individuals who scored high in self-report attentional control at pre-intervention showed the largest improvements in attention task performance. The data imply that pre-existing metacognitions concerning attention control might moderate the effectiveness of the ATT.

The effectiveness of metacognitive therapy has been tested with a range of methodologies in clinical and non-clinical participants. In their paper, Normann and Morina present a systematic review and meta-analysis of randomized trials of MCT for anxiety and depression disorders. The data appear to show that MCT is highly effective in reducing primary symptoms of anxiety or depression, secondary symptoms and hypothesized causal variables. The magnitude of effects reported seem larger than those of comparison treatments classified as cognitive-behavioral therapies. Direct comparisons of MCT with CBT in disorders such as generalized anxiety (Nordahl et al., 2018) and major depression (Callesen et al., 2020), published elsewhere, add particular weight to these results.

The special issue incorporates a series of papers reporting novel applications of MCT. Some of these are small scale or non-randomized treatment-related feasibility and acceptability papers. They are of course limited by lack of control for non-specific factors and low generalizability, but they are crucial early steps in generalizing treatment applications and offer proof of principle prior to investment in large -scale efficacy research.

Nordahl and Wells apply MCT to treating traumatized patients with Borderline Personality Disorder. This is the first evaluation of MCT with this client group, who suffer from emotion dysregulation, self-harm and impulse regulation difficulties. The study suggests that MCT is a feasible and acceptable treatment in this context. The within-group effect sizes seemed to compare favorably with the outcomes observed in other forms of therapy. Maintaining the trauma theme Simons and Kursawe conducted a feasibility study of MCT for PTSD in children and adolescents (ages 8–19 years). Treatment was associated with large improvements in PTSD symptoms and high recovery rates. The results show MCT is feasible and acceptable in traumatized youth as young as 8 years of age and justify larger scale studies.

Parker et al. explored the feasibility and acceptability of MCT in Individuals at high risk of developing psychosis. The majority of patients were able to complete treatment and gains on psychosis symptoms and secondary measures were observed. Retention at 6-month follow-up was lower and this is an area future studies should consider in the planning phase. The result is consistent with an earlier study on medication resistant patients with schizophrenia, suggesting that treatment with MCT is feasible and might be associated with significant benefit (Morrison et al., 2014). In the study reported by Caselli et al., single-case methodology was used to replicate treatment-related effects across five individuals with alcohol abuse. All patients showed clinically meaningful reductions in weekly alcohol use and number of binge drinking episodes. Winter, Naumann et al. applied MCT to adjustment disorder in a patient suffering from pulmonary arterial hypertension with noticeable gains during treatment in psychological and behavioral outcomes. The application of MCT in medical conditions is also the theme in the paper by Fisher et al.. In their study, anxiety and depression symptoms were treated in 27 cancer survivors across 6 treatment sessions. MCT appeared feasible and acceptable with 75% of patients completing the full course. Treatment appeared to be associated with large improvements in symptoms.

Exploration of the novel application of MCT not only addresses diagnoseable problems but is applied to modifying stress-related processes in the study by Myhr et al.. Here, college students who received the attention training technique (ATT) showed significant improvement in meta-worry and perceived stress compared to those that did not. The outcome indicates that ATT may reduce negative appraisal processes at both metacognitive and cognitive levels within the context of academic stress symptoms.

Most often, treatment is delivered in a one-to-one interaction between patient and therapist, but the nature of MCT, focusing on universal mechanisms, means it should be well-suited to application in groups and trans-diagnostically. These topics are addressed in three papers presented in the special issue. Callesen et al. report an uncontrolled evaluation of MCT when applied to a group of patients with a range of different diagnoses. Large pre to post treatment improvements in symptoms were observed during treatment sessions, and treatment gains appeared to be stable over follow-up.

The study by Papageorgiou et al. examined group treatment of obsessive-compulsive disorder and compared group delivered MCT with group delivered CBT. The study provides additional interest because MCT was introduced within a particular service as an attempt to improve patient outcomes beyond CBT that was traditionally offered. Whilst there was no randomization, the study is based on a benchmarking of effects of each treatment in large samples as a pragmatic evaluation of service change. CBT was associated with large improvements in OCD and related symptomatology and the effects compared favorably with those reported in the literature, but MCT was associated with better outcomes.

Several published studies have tested the effects of MCT in the treatment of GAD, and MCT is recognized in NHS NICE guidelines as a treatment option. Most often the treatment is delivered on a one-to-one-basis. Haseth et al. contribute to the group treatment literature in their feasibility study of group MCT applied to patients suffering from generalized anxiety disorder. Out of 23 consecutively referred patients 19% declined group MCT in favor of individual MCT. The group intervention was associated with a 65% recovery rate at post treatment and 78% at 3 month follow-up.

Depression is the second largest cause of global disability and a major contributor to risk through self-harm and suicide. MCT is proving that it might be a highly effective treatment for depression as shown in recent studies. Hjemdal et al. present 1-year follow-up data on their randomized trial of MCT for major depression. The results suggest a high level of maintenance of positive treatment effects following MCT, with 67% (intention to treat) and 75% of those who completed treatment classified as recovered. This is encouraging in a condition that normally has high rates of relapse. An important issue in depression treatment centers on the management of recurrent or persistent depression cases. It has been suggested that such cases require special considerations and a different treatment approach. Winter, Gottschalk et al. compared the response to MCT of patients with major depression or persistent depression. All of the persistent depression group had failed to benefit from antidepressant treatment and most of them had also received previous psychotherapy. Both sets of patients showed large and similar levels of improvement in symptoms and rates of remission during MCT and at follow-up.

Two studies in the special issue examine the question of mechanisms of change in MCT. Another study by Winter, Alam et al. capitalized on the opportunity to directly read neuronal local field signals from implanted brain electrodes in a patient with OCD during a series of MCT treatment techniques. OCD symptoms decreased after treatment and increases in alpha, beta and gamma bands and reduced theta were detected. In a different study, Johnson and Hoffart analyzed mechanism data from their earlier trial where they reported that transdiagnostic MCT was more effective than disorder-specific CBT for anxiety disorders. They found that both MCT and CBT shared some mechanisms of change; worry and attention, but additionally central to MCT was change in metacognitive beliefs about uncontrollability. Interestingly, the set of change mechanisms (in both treatments) that seemed important would be better captured by the S-REF model than by a CBT model.

Jacobsen et al. present evidence of metacognition predicting response to treatment. They delivered a brief return-to-work rehabilitation package. Whilst it was not an MCT based intervention the authors did assess metacognitive beliefs at pre-treatment and their change during treatment. Pre-treatment metacognitions were not related to return to work, but reduction in metacognitive beliefs about the need to control thoughts gave 20% greater odds of returning to work over 1 year.

Innovation in psychotherapy through the systematic use of theory-driven empiricism has been the guiding principle behind MCT development, and is a process amply demonstrated in the array of papers in this issue. The process of MCT development has eschewed the integrative and eclectic technique-driven approach in favor of developing strong theory, grounded in cognitive psychology that can inform the discovery of mechanisms of disorder and the design of specific treatment techniques. This theme is discussed in the opinion paper by Schweiger et al., in a wider context of innovation in psychotherapy. They raise important discussion questions that invite a retrospective evaluation of the barriers that have existed (and still exist in many areas) in psychotherapy evolution. They show how the process of development used in MCT offers a model that might be adopted more widely in improving psychotherapy research and treatment outcomes in the future.

## Author Contributions

AW acted as chief guest editor and conceived the Research Topic. All authors contributed to reviewing manuscripts for the special issue, acted in an editorial capacity and contributed to drafts of the editorial.

## Conflict of Interest

The authors declare that the research was conducted in the absence of any commercial or financial relationships that could be construed as a potential conflict of interest.
